# Evolution of the U.S. Biological Select Agent Rathayibacter toxicus

**DOI:** 10.1128/mBio.01280-18

**Published:** 2018-08-28

**Authors:** Edward W. Davis, Javier F. Tabima, Alexandra J. Weisberg, Lucas Dantas Lopes, Michele S. Wiseman, Michael S. Wiseman, Tal Pupko, Michael S. Belcher, Aaron J. Sechler, Matt A. Tancos, Brenda K. Schroeder, Timothy D. Murray, Douglas G. Luster, William L. Schneider, Elizabeth E. Rogers, Fernando D. Andreote, Niklaus J. Grünwald, Melodie L. Putnam, Jeff H. Chang

**Affiliations:** a Department of Botany and Plant Pathology, Oregon State University, Corvallis, Oregon, USA; b Molecular and Cellular Biology Program, Oregon State University, Corvallis, Oregon, USA; c Department of Soil Science, “Luiz de Queiroz” College of Agriculture, University of São Paulo, Piracicaba, SP, Brazil; d Department of Cell Research and Immunology, George S. Wise Faculty of Life Sciences, Tel Aviv University, Tel Aviv, Israel; e Foreign Disease-Weed Science Research Unit, USDA-ARS, Frederick, Maryland, USA; f Plant, Soil and Entomological Sciences, University of Idaho, Moscow, Idaho, USA; g Department of Plant Pathology, Washington State University, Pullman, Washington, USA; h Horticultural Crops Research Laboratory, USDA-ARS, Corvallis, Oregon, USA; i Center for Genome Research and Biocomputing, Oregon State University, Corvallis, Oregon, USA; University of Nebraska—Lincoln

**Keywords:** CRISPR, evolution, speciation, bacteriophages, plant pathogens

## Abstract

Rathayibacter toxicus is a species of Gram-positive, corynetoxin-producing bacteria that causes annual ryegrass toxicity, a disease often fatal to grazing animals. A phylogenomic approach was employed to model the evolution of R. toxicus to explain the low genetic diversity observed among isolates collected during a 30-year period of sampling in three regions of Australia, gain insight into the taxonomy of *Rathayibacter*, and provide a framework for studying these bacteria. Analyses of a data set of more than 100 sequenced *Rathayibacter* genomes indicated that *Rathayibacter* forms nine species-level groups. R. toxicus is the most genetically distant, and evidence suggested that this species experienced a dramatic event in its evolution. Its genome is significantly reduced in size but is colinear to those of sister species. Moreover, R. toxicus has low intergroup genomic diversity and almost no intragroup genomic diversity between ecologically separated isolates. R. toxicus is the only species of the genus that encodes a clustered regularly interspaced short palindromic repeat (CRISPR) locus and that is known to host a bacteriophage parasite. The spacers, which represent a chronological history of infections, were characterized for information on past events. We propose a three-stage process that emphasizes the importance of the bacteriophage and CRISPR in the genome reduction and low genetic diversity of the R. toxicus species.

## Abstract (precis)

Rathayibacter toxicus is a toxin-producing species found in Australia and is often fatal to grazing animals. The threat of introduction of the species into the United States led to its inclusion in the Federal Select Agent Program, which makes R. toxicus a highly regulated species. This work provides novel insights into the evolution of R. toxicus. R. toxicus is the only species in the genus to have acquired a CRISPR adaptive immune system to protect against bacteriophages. Results suggest that coexistence with the bacteriophage NCPPB3778 led to the massive shrinkage of the R. toxicus genome, species divergence, and the maintenance of low genetic diversity in extant bacterial groups. This work contributes to an understanding of the evolution and ecology of an agriculturally important species of bacteria.

## INTRODUCTION

Population size and standing genetic diversity affect the balance between selection and genetic drift in the evolution of species. The large population sizes of most bacterial species reduce the effects of drift, and selection is predicted to be a more dominant force (1). However, drift has a greater effect on some bacteria, mainly obligate endosymbionts and some host-restricted pathogens because bacteria with these lifestyles experience population bottlenecks upon each transmission and infection (2).

Bacteriophages have significant effects on the evolution of bacteria (3). Bacteriophages can promote the innovation of bacterial genomes via horizontal gene transfer (HGT) but can impose fitness costs and promote the diversification of bacterial genome defense mechanisms, such as the clustered regularly interspaced short palindromic repeat (CRISPR) system (4). CRISPR-associated (Cas) proteins identify and acquire target DNAs called protospacers and integrate them as spacers. This represents a form of immunologic memory in which spacers are stored in chronological order and arrayed between direct repeats (5). The array is transcribed, and spacers are processed into small RNA units that interact with Cas proteins and base pair with homologous invading nucleic acids, leading to Cas-mediated cleavage and degradation. In experimental settings, interference can provide a level of immunity that is effective in driving bacteriophage populations to extinction (6). However, in natural populations, the effectiveness of interference is attenuated by the diversity of phage populations and fluctuations in their infections (7).

There are two recognized classes and multiple subdivisions of CRISPR systems (8). Class 1, type I CRISPR systems are defined by the *cas3* gene that encodes a helicase, often fused to an endonuclease domain. The functionality of this type of CRISPR depends upon the presence of a protospacer-adjacent motif (PAM) (2 to 5 nucleotides) that is essential for acquisition and interference that is proximal to the protospacer. The type I CRISPR also engages in priming adaptation, an additional mode in which a previously assimilated spacer guides the biased and enhanced acquisition of new spacers from molecules with similar sequences (9). Priming is favored over interference when mismatches between spacer and target compromise the efficiency of interference (10, 11).

*Rathayibacter* is a genus of Gram-positive bacteria that encompasses seven species and other species that have yet to be validly described (12, 13). The ecology of these bacteria is complex, as members of *Rathayibacter* require plant parasitic nematodes of the Anguinidae taxon to be vectored to plants. Juvenile nematodes enter ovules and can induce seed galls. The nematodes can subsequently be displaced by *Rathayibacter*, which then proliferate and produce gummosis, a slime that is characteristic of the gumming diseases they cause. However, the bacteria do not always displace the nematode, and the signals that trigger bacterial growth and how *Rathayibacter* displaces the nematode are unknown.

Rathayibacter toxicus is the causative agent of annual ryegrass toxicity (12). Annual ryegrass (Lolium rigidum) was deliberately introduced as a pasture plant into Australia in 1880 (14). Historical records indicate that annual ryegrass toxicity was first reported in South Australia in 1956 and in Western Australia in 1968. The disease has been reported in more than 10 million hectares of farmland. Animals that consume infected grasses suffer episodic neurological symptoms, often leading to mortality. In Western Australia alone, it has been estimated that from 1968 to 2000, more than half a million sheep died from annual ryegrass toxicity. Toxicity is due to corynetoxin, which interferes with the early steps of protein glycosylation (15, 16). The potential severe threat to public, animal, or plant health led to the inclusion of Rathayibacter toxicus on the list of highly regulated Biological Select Agents and toxins, published by U.S. agencies in 2005 (https://www.selectagents.gov/). Its listing and its recalcitrance to genetic modification are nontrivial challenges to studying R. toxicus.

Bacteriophage NCPPB3778 is another partner in the ecology of R. toxicus (17). This phage associates with R. toxicus, and NCPPB3778 is suggested to adopt a pseudolysogenic state, an extended, arrested, and nonreplicative developmental state (17, 18). Moreover, the addition of the phage to culture-grown R. toxicus correlated with the production of corynetoxin, but genes implicated in toxin production are encoded in the genome of R. toxicus (19, 20). It is unclear whether NCPPB3778 is necessary for the synthesis of corynetoxin *in natura*, as surveys of symptomatic grass samples failed to correlate the toxin with the presence of phage (21). However, the genetic diversity of NCPPB3778 phage populations, which is uncharacterized, could have compromised conclusions that were derived on the basis of PCR detection. The genome sequence for only one strain of NCPPB3778 has been determined (18).

Isolates of R. toxicus were collected over the span of nearly three decades and from three geographic regions of Australia in which disease occurred. On the basis of similarities in patterns of amplified fragment length polymorphisms, the isolates were grouped into clusters A to C that correlated with their geographic location (22). Cluster A isolates were collected from Western Australia, and cluster B isolates were from South Australia (22, 23). The isolates of cluster C were collected from New South Wales, Australia, not South Australia as originally reported (Jim Stack, personal communication) (21). Previous results suggested that between these clusters of R. toxicus, the genomes are stable and have few detected differences between isolates. The reason for the clustering of R. toxicus into distinct groups remains unexplained, and the extent of genome stability and degree in depression of genetic diversity have not been quantified.

We used a phylogenomic approach to gain insights into the ancestry of *Rathayibacter* and to model the evolution of the R. toxicus species. We quantified gene presence/absence polymorphisms and single nucleotide polymorphisms and demonstrate that relative to other species of *Rathayibacter*, R. toxicus experienced a process of genome reduction and has exceptionally low genetic variation. A type I-E CRISPR locus was previously identified in the genome sequence of R. toxicus (20). Here, the CRISPR spacers were investigated to gain insight into the evolution of R. toxicus. Results support a proposed three-tiered evolutionary process. In the first phase, ancient and repeated infections by bacteriophage NCPPB3778 triggered population bottlenecks, large-scale gene loss, and emergence of a lineage that acquired the CRISPR locus. In the second phase, periodic selection is occurring; NCPPB3778 pressures repeatedly acting on the CRISPR locus cause recurring genome-wide sweeps that reduce population genetic diversity. Last, the separated extant R. toxicus groups are diverging, likely due to reduced migration, mutations, and genetic drift.

## RESULTS

### The *Rathayibacter* genus consists of at least nine species.

**FIG 1:**
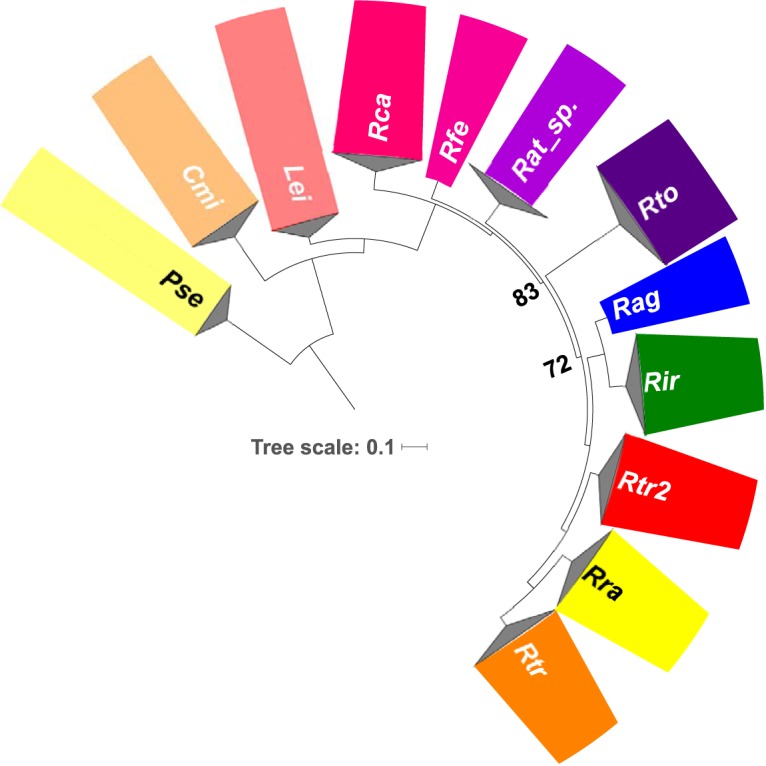
Whole-genome maximum likelihood phylogenetic tree of *Rathayibacter* species and related genera. *Rathayibacter* is a distinct phylogenetic unit comprised of nine species-level groups. The phylogenetic tree is based on all single-copy orthologous genes. Genera were collapsed. Bootstrap values are all 100% except for those reported. The groups are color coded based on species (*Rathayibacter* species) or broader group (*Pseudoclavibacter*, *Clavibacter*, and *Leifsonia*). Species and genus abbreviations: *Rca*, R. caricis; *Rfe*, R. festucae; *Rat*, *Rathayibacter* species; *Rto*, R. toxicus; *Rag*, R. agropyri; *Rir*, R. iranicus; *Rtr2*, R. tritici-like; *Rra*, R. rathayi; *Rtr*, R. tritici; *Pse*, *Pseudoclavibacter*; *Cmi*, *Clavibacter*; *Lei*, *Leifsonia*. Bar, 0.1 nucleotide substitutions per position.

We determined the genome sequences for 112 isolates of *Rathayibacter* (see Data Set S1A in the supplemental material). Twenty-two of the isolates are members of R. toxicus clusters A, B, and C (hereafter referred to as groups or clades) collected from three regions in Australia (22). Another 71 isolates were sampled over a 4-year period from grasses growing in 10 counties in the state of Oregon in the United States. The remaining isolates, many of which were from culture collections, were selected for their representation of taxonomic units of *Rathayibacter*. The assembled genomes ranged from 2.3 to 4.4 Mb in size, and the G+C content ranged from 61.5% to 72.7%. A maximum likelihood phylogenetic tree was constructed on the basis of all single-copy orthologous gene sequences. The *Rathayibacter* genus formed a distinct clade that is sister to *Leifsonia* (Fig. 1; all nodes had bootstrap values of >70%).

10.1128/mBio.01280-18.4 DATA SET S1  Supplemental data sets showing metadata of sequenced *Rathayibacter* isolates (Data Set S1A), average nucleotide identity (ANI) values of all possible pairwise comparisons of isolates of *Rathayibacter* and related genera (Data Set S1B), predicted functions of core and enriched genes of *Rathayibacter* (Data Set S1C), pairwise differences in single nucleotide polymorphisms (SNPs) for *R*. *toxicus* and *R*. *tritici*-like isolates (Data Set S1D), *R*. *toxicus* genes with evidence of positive selection (Data Set S1E), predicted functions of core genes and genes unique to *R*. *toxicus* (Data Set S1F), unique spacers of *R*. *toxicus* (Data Set S1G), and accession numbers of genome sequences used in this study (Data Set S1H). Download DATA SET S1, XLSX file, 0.3 MB. Copyright © 2018 Davis et al. 2018 Davis et al. This content is distributed under the terms of the Creative Commons Attribution 4.0 International license.

Within *Rathayibacter*, there are nine clades comparable to species. To confirm this, we calculated and analyzed all pairwise average nucleotide identity (ANI) values between genome sequences (see Fig. S1 and Data Set S1B in the supplemental material) (24). Within the *Rathayibacter* genus, intraclade diversity is low, with most pairwise ANI values exceeding 99%. Interclade comparisons ranged in ANI values from 72% to 90%, with the lowest values obtained in pairwise comparisons to R. toxicus. On the basis of a threshold of ≥94% ANI, we categorized the isolates into nine units. Seven are validly named species. Another two units, Rathayibacter  tritici-like (*Rtr2*) isolates and unnamed isolates of *Rathayibacter* (Leaf185/294) were operationally classified as new species. Of the 71 isolates sampled from Oregon, 20 isolates were classified as R. rathayi and 51 were classified as *Rtr2*. According to analysis of ANI, *Rtr2* isolates form two subclusters that are close to the threshold for differentiation into two species groups and if we had used a threshold of ≥96% would have classified them as separate species (Data Set S1B). Instead, we elected to consider them as *Rtr2* clades A and B and focused on the more represented clade A. To disambiguate between clades of R. toxicus and *Rtr2*, those of the latter will be specified via use of parenthetical qualifiers, e.g., *Rtr2* (clade A). Some of the newly defined clades of *Rathayibacter* are represented by few isolates and require deeper sampling for increasing confidence in species identities. For instance, using a higher ANI threshold, Rathayibacter caricis could represent multiple species groups, and R. rathayi could represent two species groups.

10.1128/mBio.01280-18.1 FIG S1  *Rathayibacter* circumscribes nine species. Average nucleotide identity (ANI) was used to analyze all possible pairwise comparisons of genome sequences (see Data Set S1B for values). Species-level groups were defined based on an ANI threshold of 94% ANI cutoff. Three-letter codes are identical to those used in Fig. 1. The color key (bottom left) relates colors to the range of ANI values. Download FIG S1, EPS file, 0.8 MB. Copyright © 2018 Davis et al. 2018 Davis et al. This content is distributed under the terms of the Creative Commons Attribution 4.0 International license.

### R. toxicus evolved via genome reduction.

**FIG 2:**
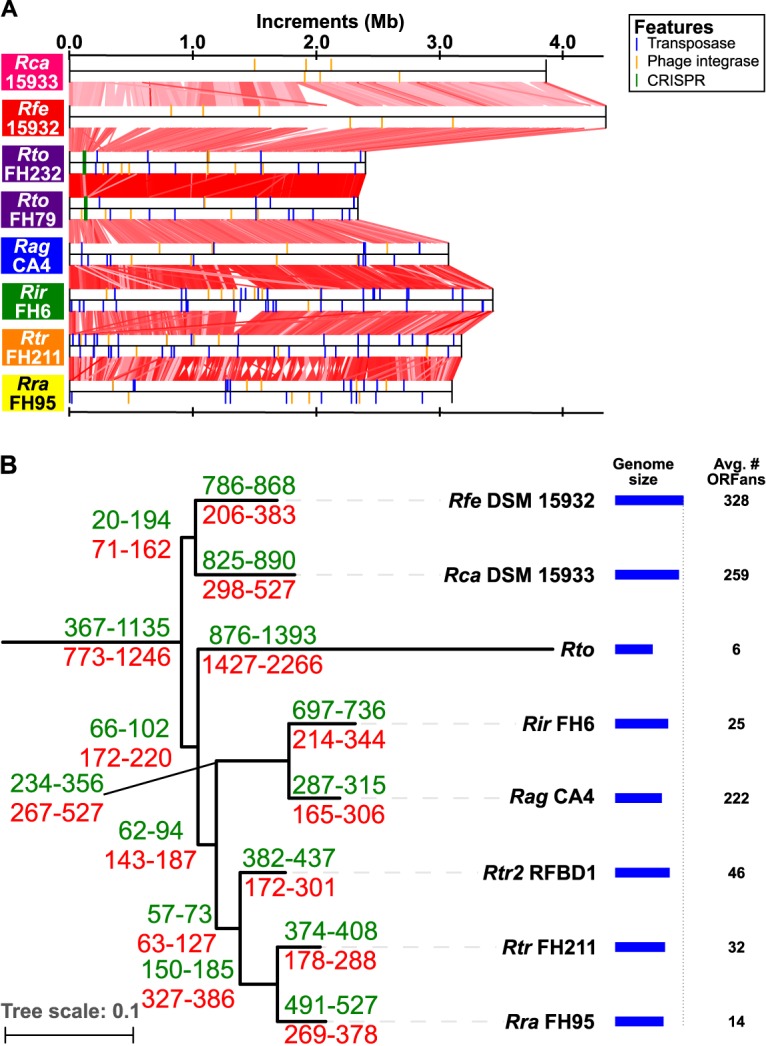
Comparison of *Rathayibacter* genomes. (A) The *Rathayibacter* genomes are similar in structure. Colinear blocks are indicated with red lines and shaded according to percent identity (50% identity indicated by lightest pink; 99.5% identity indicated by darkest red). Colored vertical lines are used to indicate the locations of select genome features (see legend). (B) Reconstruction of gene gains (green) and losses (red) for *Rathayibacter*. ** **Reconstruction was iterated in which isolates representing the different clades of R. toxicus were tested to identify the minimum and maximum predicted gene gains and losses. Genus level comparisons were between *Rathayibacter* and *Leifsonia*. The blue bars indicate the relative genome sizes of isolates that represent each of the taxonomic units (scaled to DSM 15932). The average number of predicted orphan genes (ORFans) per taxonomic unit is shown. Species abbreviations: *Rca*, R. caricis; *Rfe*, R. festucae; *Rto*, R. toxicus; *Rag*, R. agropyri; *Rir*, R. iranicus; *Rtr*, R. tritici; *Rra*, R. rathayi; *Rtr2*, R. tritici-like.

The long branch leading to R. toxicus and the high interclade divergence in ANI are consistent with an accelerated rate of evolution in this species relative to others in the genus (Fig. 1 and Fig. S1). Furthermore, the genome of R. toxicus is significantly smaller in size than those of other species of *Rathayibacter* (~2.3 Mb in size; *P* value ≤ 0.0001 in a likelihood ratio test; Fig. 2 and Data Set S1A) and has the lowest G+C content of 61.5%. To further examine evolutionary differences, we characterized the large-scale changes of eight genomes that were sequenced using PacBio or 454 technologies and assembled into few contigs (Data Set S1A) (20). Despite the significant decrease in relative size, the R. toxicus genomes of isolates FH232 and FH79 belonging to clades C and A, respectively, have few large-scale rearrangements relative to each other (21) (Fig. 2A). Clade B was not analyzed because no member was sequenced using PacBio or 454 technologies. In comparison to genomes of some species, the genomes of isolates from clades C and A of R. toxicus have one or two inverted regions flanked by genes encoding tRNAs or integrases, but the blocks are otherwise colinear (Fig. S2). Analysis of cumulative GC skew shows that the inverted regions encompass replication terminators. The plots of GC skew also show that R. toxicus has the most easily defined origin of replication and terminator as well as the most balanced replichores, relative to those of other species being compared (Fig. S2). We found few sequences with homology to mobile genetic elements in the genome sequences that were determined using PacBio or 454 technologies. Rathayibacter tritici FH211 had the most transposase-encoding genes, with 75 of these genes. Only one other genome sequence exceeded 50 putative transposase-encoding genes. We also identified in these genome sequences an ~75-nucleotide-long palindromic repeat sequence in the genomes of *Rathayibacter*. Rathayibacter festucae has 99 copies of the repeats, whereas other isolates varied from 15 to 50; R. toxicus has 19.

10.1128/mBio.01280-18.2 FIG S2  The genomes of *Rathayibacter* are structurally similar. (A) Whole-genome alignment. Genomes were ordered such that *dnaA* is the first gene. Mauve was used to construct the multiple genome alignment. The colors indicate local colinear blocks. Transparent red rectangles outline the regions that are inverted in genomes of *Rathayibacter*. Red vertical lines in the alignment of the *Rag* genome represent contig breaks. (B) Cumulative GC plot for representative *Rathayibacter* genomes. Plots show GC skew calculated for 5-kb sliding windows (black lines), and cumulative GC skew values (orange lines), with global maximum values corresponding to the terminus (vertical red lines) and minimum values corresponding to the origin of replication (vertical green lines). Vertical blue lines demarcate borders of inverted regions. *Rag* was not analyzed because its genome is in three fragments. Abbreviations: *Rca*, R. caricis; *Rfe*, R. festucae; *Rto*, R. toxicus; *Rag*, R. agropyri; *Rir*, R. iranicus; *Rtr*, R. tritici; *Rra*, R. rathayi. Download FIG S2, EPS file, 2.5 MB. Copyright © 2018 Davis et al. 2018 Davis et al. This content is distributed under the terms of the Creative Commons Attribution 4.0 International license.

We next examined the evolutionary dynamics of gene gains and losses in *Rathayibacter* (Fig. 2B). In the lineages leading to R. rathayi, R. tritici, R. tritici-like, R. iranicus, and R. agropyri, which form a sister clade to R. toxicus, there were more predicted ancient gene losses than gains. However, each of these species groups is predicted to have more recent gene gains than losses. In contrast, R. toxicus is characterized by an approximately twofold enrichment in losses than gains (Fig. 2B). In fact, the estimated number of predicted gene losses in R. toxicus is higher than the number predicted between the two sister genera *Rathayibacter* and *Leifsonia*. When we focused on only the sequenced isolates of the five species that share a recent common ancestor with R. toxicus, there are 1,029 genes predicted to be core genes. Of these genes, 336 had no identifiable ortholog in the genome sequence of even a single sequenced isolate of R. toxicus, suggesting that their absence reflected genuine gene loss events (Data Set S1C). We used a Welch’s *t* test to identify 345 orthologous clusters with genes that are significantly enriched in presence in the genomes of *Rathayibacter* relative to those of closely related genera (Data Set S1C). Genes that are enriched in most species of *Rathayibacter* likely have functions associated with specific adaptations of the genus. A subset of 110 genes are absent in R. toxicus, 63% of these genes are annotated as hypothetical, and the functions and implications of their loss from R. toxicus are unknown.

Consistent with having a reduced genome, R. toxicus has the lowest G+C content within the genus (2). Decreases in G+C content are often associated with loss of DNA repair pathways. R. toxicus, like all members of *Rathayibacter* and many *Actinobacteria*, lacks homologs of *mutS*, necessary for mismatch repair. However, all examined members of *Rathayibacter* have homologs of the noncanonical mismatch repair pathway (25). All examined genome sequences carry genes that encode components of repair pathways such as homologous recombination, base excision repair, and nucleotide excision repair. The *ku* and *ligD* genes necessary for a nonhomologous end joining pathway in *Mycobacterium*, *Bacillus*, and a few other taxa of bacteria are also present in the *Rathayibacter* genus (26). However, R. toxicus lacks homologs of both *ku* and *ligD*, whereas R. rathayi lacks *ku* and isolate FH6 of R. iranicus has a frameshift in *ligD*. Therefore, changes in nucleotide composition in R. toxicus are not likely a consequence of loss of function in DNA repair pathways.

**FIG 3:**
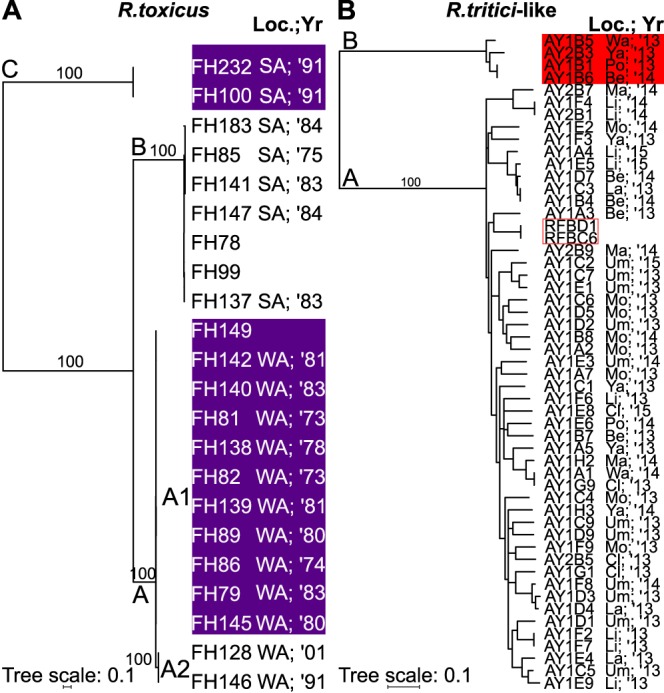
R. toxicus and R. tritici-like isolates form genetically distinct clades. Maximum likelihood phylogenetic tree constructed on the basis of pairwise genome SNP differences in R. toxicus (A) and R. tritici-like isolates (B). The locations (Loc.) where the isolates were collected and years (Yr; two-digit abbreviations) are shown. The isolates were collected in Australia (SA, South Australia; WA, Western Australia) and Oregon (Be, Benton; Cl, Clackamas; La, Lane; Li, Linn; Ma, Marion; Mo, Morrow; Po, Polk; Um, Umatilla; Wa, Washington; Ya, Yamhill). If the location or year is unknown, it is left blank. The RFBD1 and RFBC6 (red box) were sequenced in the first effort. Branches with bootstrap support of <50% were collapsed; values for key branches are shown. The purple and red blocks are used to help visualize the different clades and subclades.

Individual isolates of R. toxicus had few unique gene gains subsequent to the emergence of the species. R. toxicus is predicted to have the smallest average number of orphan genes (Fig. 2B). A rarefaction curve was used to analyze the pan-genomes for R. toxicus and its groups. The pan-genome of the species is technically open but growing slowly (γ = 0.053; 0 < γ < 1 indicates an open pan-genome), while the estimated core genome of 1,985 genes represents an average of 90% ± 1% of each genome (27, 28). Estimates of pan-genome and core genome sizes were also calculated for groups A1 and B, which had a sufficient number of isolates for the analysis (see next section and Fig. 3A for explanation of group A1). The γ values (A1 = B = 0.016) are even closer to zero. The representation of the core genomes of 2,125 and 2,124 genes ranges between 95.5% to 96.8% and 96.2% to 96.7% of the genomes of their respective members. Last, we used GI_SVM, which identifies regions distinct on the basis of compositional bias of *k*-mers, to determine the contribution of HGT (29). We reasoned that horizontally acquired regions should either be specific to the lineage in which it was identified or homologous only to regions also identified by GI_SVM. There was an average of 20.5 ± 3.4 regions (≥5 kb) per genome that were predicted to differ in compositional bias of *k*-mers. However, all regions are homologous to at least one region in another genome sequence that GI_SVM did not identify. These three methods yielded results consistent in supporting the conclusion that the occurrence of HGT after the formation of the R. toxicus species is low.

### R. toxicus forms genetically homogenous groups.

The R. toxicus species forms three clades, as determined on the basis of a whole-genome single nucleotide polymorphism (SNP) tree (Fig. 3A and Data Set S1D) (22, 23). Within clades A and B, the average numbers of pairwise SNP differences defined on the basis of comparisons to a single common reference genome sequence of isolate FH232 are 46 ± 70 and 7 ± 4, respectively. In clade A, two of the isolates have 156 ± 5 pairwise SNP differences relative to other members of the clade. We therefore recognized subclade A2 (isolated in 1991 and 2001; three pairwise SNP differences) as temporally separated from subclade A1 (isolated from 1973 to 1983; 2 ± 2 average pairwise SNP differences). Given an average genome size of 2.32 Mb for R. toxicus, there are fewer than three pairwise SNPs per Mb for the subclades of A and clade B. The average number of pairwise SNP differences per megabase between the A, B, and C clades exceeds 800.

To test whether low genetic diversity is unique to R. toxicus, we used *Rtr2* (clade A) as a comparator group. This group was selected because it was sufficiently sampled for our analyses (Data Set S1A; see subsampling below). *Rtr2* (clade A) is substantially more heterogenous than the clades of R. toxicus. In the SNP tree, *Rtr2* (clade A) is partially resolved with some polytomies (Fig. 3B). *Rtr2* (clade A) has an open pan-genome (γ = 0.259) and a core of 2,213 genes that represents between 63% to 70% of the genomes of the 47 members. There is an average of 912 pairwise SNPs per Mb, defined on the basis of comparisons to a single common reference genome sequence of isolate RFBD1, within *Rtr2* (clade A) (Data Set S1D).

**FIG 4:**
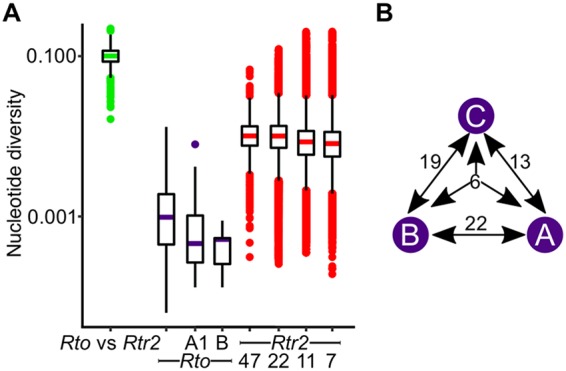
Genetic diversity in R. toxicus groups. (A) Whisker box plots representing the nucleotide diversity (π) for 920 genes with orthologs in all sequenced isolates of R. toxicus (*Rto*; 22 total isolates, with 11 and 7 isolates in subclades A1 and B, respectively) and clade A of R. tritici-like isolates (*Rtr2*; 47 isolates). For the subsets of *Rtr2* isolates, plots are the totals of 100 resampled isolates. The confidence intervals were 0.012 to 0.0136 (22 isolates), 0.008 to 0.012 (11 isolates), and 0.008 to 0.013 (7 isolates). (B) Diagram showing the number of genes with evidence for positive selection in all possible comparisons between clades A to C (purple circles) of R. toxicus.

A total of 920 single-copy orthologous genes present in all members of R. toxicus and *Rtr2* (clade A) were characterized. Within this set, the genes of R. toxicus (π = 0.0011) have a 10-fold-lower average nucleotide diversity than those of *Rtr2* (clade A) (π = 0.011) (Fig. 4A). The distribution of nucleotide diversity value per gene is heavily skewed toward 0 in R. toxicus, while the distribution for *Rtr2* (clade A) is more normally distributed, with a peak near 0.01. We confirmed, by subsampling 100 times, 22, 11, and 7 randomly selected isolates of *Rtr2* (clade A) to match the sizes of the R. toxicus groups, that differences are not due to variations in sample size (Fig. 4A). Of the 920 R. toxicus orthologous genes, 29% have no polymorphisms when examined at the level of the species. When examined within clades A1 and B, 96% and 99%, respectively, of the genes have no polymorphisms. In contrast, *Rtr2* (clade A) has no monomorphic genes, no matter the sample size examined.

We next examined the 920 genes for evidence of allelic fixation in each of the three clades of R. toxicus. A total of 216 orthologous genes were identified; the nucleotide diversity of these genes was zero in any of the three clades of R. toxicus and nonzero in all of R. toxicus. Of these genes, 60 genes had a ratio of nonsynonymous to synonymous substitutions (*dN*/*dS*) of >1, which is often used as evidence for positive selection (Fig. 4B and Data Set S1E). However, all 60 genes are apomorphies, as no fixed allele was identified in more than one pairwise comparison between clades of R. toxicus. In addition, although six genes had a *dN*/*dS* value of >1 when all three clades were compared, the genes have multiple nucleotide differences, and each clade is fixed for a unique allele. Sixteen orthologous genes have multiple differences, and 12 have at least one pair of nucleotide difference that is within 150 nucleotides of each other, potentially reflecting recombination. Last, 54 of the translated coding sequences have an assigned function. These data are not consistent with divergent selection acting on the 60 fixed alleles.

### The isolates of R. toxicus have type I-E CRISPR loci enriched for spacers against NCPPB3778.

**FIG 5:**
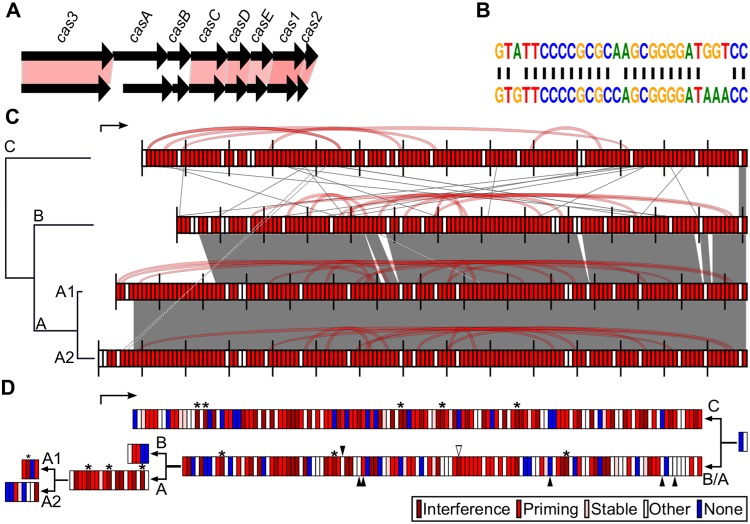
R. toxicus isolates encode a type I-E CRISPR-Cas system. (A) The structure of the *cas* locus of R. toxicus (top) compared to that of the *cas* locus of E. coli K-12 (bottom). Red blocks highlight genes with homologous translated sequences (27% identity indicated by the lightest color and 40% identity indicated by the darkest color). (B) Alignment of the direct repeat sequence from the R. toxicus CRISPR array (top) to the direct repeat sequence from the E. coli CRISPR array (bottom). (C) Structures of the four CRISPR arrays, mapped to a cladogram of R. toxicus. The spacers are arranged from the most recent (left) to the most ancient (right). The arrow represents the direction of transcription. Spacers shaded in red are predicted to target bacteriophage NCPPB3778, as detailed in panel D. Spacers that are similar between CRISPR arrays are connected via gray lines; spacers of clade C similar to those of clades A1 and A2 are connected via dotted gray/white lines. Spacers that are similar within a CRISPR array are connected via red lines. (D) Functional categorization and chronology of CRISPR spacer acquisition. The four different CRISPR arrays were collapsed on the basis of their relationship. The spacers are arranged from the most recent (left) to the most ancient (right). The single-headed arrow shows the direction of transcription. The double-headed arrows indicate the points in which the CRISPR arrays diverged. Asterisks indicate that the spacer is identical to the protospacer in the extant NCPPB3778 sequence. Solid black triangles indicate deletion of the spacer in all members of one clade. The white triangle indicates deletion of the spacer in one isolate of clade B.

R. toxicus has 181 orthologous clusters that represent core genes and genes that are unique to the species (Data Set S1F). The genes were likely horizontally acquired by the ancestor that led to R. toxicus, and the genes have subsequently been vertically inherited by extant members of the species. More than 60% of the 181 genes unique to R. toxicus are annotated as encoding hypothetical proteins. Of the others, the most distinguishable predicted functions are associated with the type I-E CRISPR-associated proteins (Fig. 5A) (20). In R. toxicus, there are only 58 nucleotides between *cas2* and the most recently acquired spacer. Leader sequences are typically between 81 and 599 nucleotides long, though sequences as short as 60 nucleotides have been shown experimentally to be sufficient (30, 31). The CRISPR arrays of R. toxicus have 131 to 149 spacers, which is approximately double the average number of spacers present in CRISPR loci previously analyzed (32). The direct repeat sequence between spacers in R. toxicus is similar to the cluster 2 repeats of CRISPR arrays in Escherichia coli (Fig. 5B) (33). Bases predicted to form the stem of the stem-loop are the most conserved. The two most ancient spacers are identical in all CRISPR arrays and flanked by degenerate direct repeat sequences (34).

We used spacer composition to cluster the CRISPR arrays into four sets and mapped the sets to a cladogram of R. toxicus (Fig. 5C). The CRISPR array in clade C is the most divergent but includes spacers that are similar to those in the other arrays. For example, spacer 126 of clade C and spacer 34 of clades A and B have identical sequences, and spacer 116 of clade C and spacer 62 of clades A and B have similar sequences. The majority of the spacers present in members of (sub)clades A1, A2, and B are identical and arrayed in the same order. There are a few minor exceptions. Between CRISPR arrays of clades A and B, there are six internal deletions (Fig. 5D). Within clade A, the four most recently acquired spacers in subclade A1 and eight spacers in subclade A2 are variable (Fig. 5C). The composition of CRISPR spacers within isolates of (sub)clades A1, A2, and B, is conserved. The only exception is the deletion of a single spacer from the CRISPR array of isolate FH147 in clade B (Fig. 5D). The conservation of more ancient spacers and deletion of spacers have been previously reported (35). Our findings are consistent with historical records, which suggest that R. toxicus migrated between 1956 and 1968 from South Australia (group B) to Western Australia (group A). Our findings also validate the classification of subclades A1 and A2 into temporally separated groups.

We wrote a computer algorithm to bin the R. toxicus spacers into one of five categories (Fig. S3A). The rules used for binning were derived from analyses of an empirically derived data set (10). Strikingly, 53% of the 300 unique spacers were predicted to function in interference and priming against bacteriophage NCPPB3778 (Fig. 5C and D). The 159 unique spacers predicted to target NCPPB3778 are distributed throughout the CRISPR arrays and mapped throughout the bacteriophage genome sequence (Fig. 5D and Fig. S3B). After divergence of arrays associated with clades A and B, the clade A-associated array gained 13 spacers, and the array of clade B gained 2 spacers that target NCPPB3778. Between subclades A2 and A1, the arrays vary by the 12 most recently acquired spacers of which 5 are predicted to target NCPPB3778. Of the 67 spacers predicted to provide interference to the extant NCPPB3778, 34 spacers from clade C are distributed evenly throughout the array. In clades A and B, the distribution of 33 interference spacers is biased toward more recent acquisitions.

10.1128/mBio.01280-18.3 FIG S3  Rules implemented in the algorithm to predict functional outcomes of spacer-protospacer interactions. (A) Schematic for the crisprhit algorithm. Spacers were binned into one of five categories: direct interference (perfect and high quality), elimination of target; priming, acquisition of new spacers to target; stable, spacer is ineffective in targeting; unknown (other), weak signal, no prediction. Binning was completed on the basis of a five-stage filtering process (middle; gray rectangles). A test data set was analyzed in a proof-of-concept experiment (10). Each stage is associated with a decreasing amount of confidence (increasing number of mismatches between a spacer and protospacer). Colored lines represent the relative amounts of spacers that were filtered (few [thin lines] to many [thick lines]) (there are few perfect matches because the test data set consisted of variant sequences) into each of the interaction categories. Vertical arrow-tipped lines each represent ~2.5% of the spacer-protospacer interactions from the test data set, and the arrows thus represent the percentage of spacers that meet each filter (percentages are listed to the right of each filter). General rules used as filtering criteria are shown in the key (10). (B) Spacer-protospacer interactions predicted for all nonredundant spacers encoded in R. toxicus. White arrows depict predicted coding sequences of NCPPB3778. Colored lines show position and strand (arrows above for plus strand and arrows below for minus strand) of putative spacer-protospacer interactions. The crisprhit algorithm was used to predict the types of interactions, and results are color coded accordingly (see key). Download FIG S3, EPS file, 0.1 MB. Copyright © 2018 Davis et al. 2018 Davis et al. This content is distributed under the terms of the Creative Commons Attribution 4.0 International license.

We identified pairs of spacers with slight differences in sequences within arrays; these pairs of spacers were nonetheless predicted to target the same regions of NCPPB3778 (Fig. 5C and Data Set S1G). For example, spacer 30 from clade C is 1 nucleotide longer than spacer 60, and both spacers are predicted to be high-quality interference spacers that target the same strand. Spacers 6 and 68, also from clade C, differ by 5 nucleotides and are predicted to cause priming. Spacers 48 and 67 of clades A and B (numbered according to the position in arrays of clade B) are predicted to be “other” and high-quality interference, respectively. These spacers differ by 1 nucleotide in length, but they are also polymorphic at three other positions. Last, spacers 40 and 145, common to clades A and B, differ by 6 nucleotides and are predicted to be “other” and sufficient for priming, respectively. The recurrence of spacers with similar sequences is indicative of diversity within phage populations.

No spacer sequences had identifiable homology to any sequence other than NCPPB3778. Spacers with no homology were classified as “none” and are hypothesized to target yet-to-be identified bacteriophages or plasmids of R. toxicus. Searches for homologs of anti-CRISPR proteins in NCPPB3778 failed to reveal candidates (36).

## DISCUSSION

To contribute to a framework for studying *Rathayibacter*, we generated finished and draft genome sequences from members that represent the diversity of the genus. A phylogeny, coupled to whole-genome analyses, supported the existence of at least nine species. We focused on gaining insights into the evolution of R. toxicus because as the causative agent of annual ryegrass toxicity, this species has had significant impact in Australia and threatens agricultural industries of other countries (12). Our results support the possibility of three phases in the evolution of R. toxicus.

In the first phase, an ancient lineage susceptible to bacteriophage NCPPB3778 is hypothesized to have suffered repeated and massive losses to its genome. NCPPB3778 adopts a pseudolysogenic state, which could lead to long-term coexistence with increased frequency in lytic cycles and repeated bottlenecks to the host population (17). The periodic resetting of diversity to near zero is highly disruptive to the process of HGT. Reduced frequency of gains, coupled to the inherent deletion bias of bacterial genomes, will result in genome attrition and yield a structurally stable genome that, relative to sister species, has few large-scale rearrangements and is richly punctuated by gene deletions (Fig. 2A and see Fig. S2 in the supplemental material) (37). Genome reduction of R. toxicus must have occurred prior to the acquisitions of the third most ancient spacers (Fig. 5). Thus, genome attrition likely occurred previous to, or concurrent with, the period during which the CRISPR locus was acquired. We further speculate that intense pressure by NCPPB3778 could have selected for the rare genotype that acquired the CRISPR locus.

Genome reduction is often associated with a change toward an obligate endosymbiont lifestyle (2). However, R. toxicus can be cultured on a standard medium, and inspection of the annotated functions of its genome sequences did not reveal any evidence of these bacteria being dependent on a host for survival (20).

In the second phase in the evolution of R. toxicus, the extant species carrying a CRISPR locus evolves via periodic selection, a recurring process of adaptive changes and genome-wide selective sweeps (38). This is consistent with theory and observations from metagenomic reconstructions showing that phage blooms are a periodic force that act on CRISPR loci causing rapid selective gene sweeps and conservation of trailing end spacers (7, 35, 39). Isolates FH128 and FH146 (1991 to 2001) of subclade A2 potentially represent the emergence of a new dominant immune genotype (Fig. 3A and 5B). In 2013 to 2014, another genetically distinct group with low genetic diversity emerged and dominated the sites surveyed in South Australia (23). Recent studies have provided evidence that periodic selection has the potential to occur in complex ecosystems (40, 41).

It has been suggested that recombination and promiscuous exchange of DNA are barriers to divergence (42, 43). Our observations provide one possible explanation to resolve this conflict. A high frequency of phage blooms reduces genetic heterogeneity of populations, dampening the effects of recombination and deterring HGT. It has been suggested that CRISPRs directly hinder HGT, but CRISPRs have no discernible effects when viewed on a larger evolutionary time scale (44–46). When coupled to frequent bottlenecks, however, the impacts of CRISPRs could be magnified.

In the third phase, the R. toxicus groups are diverging (Fig. 3 and 4). Our model predicts a single introduction or emergence in Australia and the early divergence of clade C from the most recent common ancestor of the three clades that were sequenced. Between 1956 and 1968, R. toxicus migrated from South Australia to Western Australia, and established a new population represented by clade A. NCPPB3778 bacteriophage comigrated, as results show continual and local adaptive effects on the CRISPR locus of group A (Fig. 5). These local effects are consistent with conclusions that spatial structuring explains the observed diversity at CRISPR loci (47, 48).

Between the groups of R. toxicus, there are more than 800 pairwise SNPs, and of 920 loci examined, 216 loci are fixed within each clade (Data Set S1D). Sixty loci have a *dN*/*dS* value of >1, and it is possible that one or several of these loci provide a local selective advantage (Fig. 4 and Data Set S1E). However, despite sharing a very recently derived common ancestor, among the 60 genes, there are no alleles common to groups A and B, relative to group C. This is not consistent with loci being under positive selection and providing a fitness benefit. Moreover, 54 of the 60 loci are assigned a function, and the proteins do not provide fitness benefits we can predict on the basis of their annotations. Finally, of the 44 orthologs with a single nucleotide difference, 36% were predicted to have conservative amino acid differences, which may have little effect on the function of the protein. Because strong selection pressures by NCPPB3778 frequently act on CRISPR loci and impose severe bottlenecks to R. toxicus, our leading hypothesis is that apparent fixation of alleles under divergent selection is a consequence of random genetic mutations and whole-genome hitchhiking in the most immune genotypes. We further suggest that migration between groups must be low for the groups to exhibit local variation. Management strategies implemented to control annual ryegrass toxicity could explain the separation between groups of R. toxicus.

Sampling bias is not likely an explanation for the low diversity in R. toxicus groups because the geographically separated isolates showed similar patterns, and isolates were collected over the span of decades from multiple plant host species, as well as by different researchers (Data Set S1A) (22). We also demonstrated that the genetic diversity of R. toxicus was substantially less than the diversity in R. tritici-like (*Rtr2*) (clade A) isolates that were collected over a significantly shorter period of time and in a more geographically restricted location (Fig. 4). However, while the sequencing strategy reveals the most dominant genotypes in Australia, it fails to reveal the frequency at which rarer genotypes occur within the groups.

Despite the CRISPR immunity, bacteriophage NCPPB3778 continues to exert a strong influence on R. toxicus. There are several factors that could contribute to the coexistence of the antagonistic partners. Previous findings showed that populations mixed with genotypes encoding diverse spacers cause rapid declines in phage persistence (6). However, R. toxicus is not expected to drive NCPPB3778 to extinction because although the spacers are numerous and diverse, there is little within-population diversity. Phage genomes are highly mosaic, and diversity allows subpopulations to evade CRISPR immunity (7, 49). The extent of the diversity of NCPPB3778 is unknown, but given the proliferation of spacers that target it and the evidence for priming, we suggest that the phage population is genetically divergent. Because each of the four unique arrays has ~20 spacers with no homology to the NCPPB3778 sequence (classified as none), it is possible that a different type of phage selects for bacterial genotypes that are less immune to NCPPB3778. Stochastic effects are important because when high numbers of infections occur, phage will inevitably evade immunity (5). Pseudolysogeny may help NCPPB3778 resist detection by the CRISPR system, which shows preference toward replicating molecules (50). Pseudolysogeny is also an indicator of a nutrient-poor condition, one that compromises immunity (51).

Results presented in this study underscore the importance of phage in the evolutionary and population dynamics of bacteria. NCPPB3778 and the CRISPR immune system are hypothesized to drive the evolution of R. toxicus, which is consistent with recent findings implicating CRISPRs in speciation of bacteria (52). Whether the low genetic diversity compromises the ability of R. toxicus to migrate from Australia and adapt to new environments is unknown. Within Australia, there is evidence that the extant populations are diverging. The data set described here provides a phylogenetic and genomic framework for future studies on this important and unusual genus of plant-associated Gram-positive bacteria.

## MATERIALS AND METHODS

### Bacterial growth.

*Rathayibacter* cells were cultured from seeds collected from 2012 to 2015 and obtained from the Oregon State University Seed Laboratory. Four grass species (Lolium perenne, Lolium multiflorum, Agrostis stolinifera, and Dactylis glomerata) were collected from 13 counties in the state of Oregon. Each seed lot was washed for 24 h at 4°C in 1:10 (wt/vol) physiological saline containing 2% cycloheximide. Supernatant from each sample was streaked to four plates of medium D2, and plates were incubated at 28°C (53). Colonies were evaluated on the basis of morphology and via Gram staining, approximately 11 days and 21 days after streaking. Colonies were randomly selected from those colonies with morphology consistent with *Rathayibacter* that had stained positively. Bacteria were grown in liquid lysogeny broth and shaken at 28°C until turbid (54).

### DNA sequencing and assembling.

To prepare DNA for Illumina sequencing, DNA was extracted using the DNeasy blood and tissue kits (Qiagen, Valencia, CA), following instructions for Gram-positive bacteria. A NanoDrop ND-1000 UV-visible (UV-Vis) spectrophotometer and Qubit 2.0 fluorometer (Thermo Fisher Scientific, Waltham, MA, USA) were used to measure the quality and quantity, respectively, of the DNA. Libraries were prepared, following the Illumina Nextera XT library preparation protocol and sequenced on the Illumina HiSeq 3000 system (Illumina Inc., San Diego, CA, USA) by the Center for Genome Research and Biocomputing (CGRB) at Oregon State University. Previously reported methods were followed to process, assemble, and annotate sequencing reads and contigs (55).

To prepare DNA for PacBio or 454 sequencing, a modified Marmur method was used (56). Sequencing libraries were either prepared for the PacBio RSII system (Pacific Biosciences, Menlo Park, CA, USA) or for the 454 Junior system (Roche, Branford, CT, USA) according to the manufacturer’s directions. PacBio sequencing was done by the Genomics Lab at Washington State University, whereas three or four 454 sequencing runs per strain were performed in-house at the USDA-ARS Foreign Disease-Weed Science Research Unit (FDWSRU). The hierarchical genome assembly process (HGAP) was used to assemble genome sequences (57).

Mauve v. 2.4.0 and the finished sequences were used to order contigs (58).

### Analyses of whole genomes.

Pairwise average nucleotide identities (ANIs) were calculated using autoANI and according to the methods previously described (59, 60).

A restricted maximum likelihood test (brownieREML), implemented in the phytools R package, was used to generate likelihood values for a likelihood ratio test (df = 1) of the significance of the reduction in genome size of R. toxicus relative to the genome sizes of other *Rathayibacter* species (61).

ProgressiveMauve and lastz were used to generate whole-genome alignments (58, 62). The SeqUtils module of biopython was used to calculate GC skew (5.0-kb sliding windows) (63). Lastz alignments and GC skew were plotted using the GenomeDiagram module of biopython (63).

Bowtie2 v2.3.3.1 was used to align sequencing reads from isolates of R. toxicus and R. tritici-like to the corresponding reference sequences of R. toxicus FH232 or R. tritici-like RFBD1 (64). Single nucleotide polymorphisms (SNPs) were identified using the haplotype caller of the genome analysis toolkit (GATK) software (65). VCFR was used to filter for high-quality SNPs (66). To be included in the final analysis, the nucleotide positions had to meet the criteria of having a read depth of 25% to 75% of total reads of the corresponding genome and of being present in ≥80% of the genome sequences.

We used GET_HOMOLOGUES v. 2.0 and the OrthoMCL algorithm (-M), with a minimum sequence identity cutoff of 35% (-S 35) to generate clusters of orthologous proteins (67). To determine the functional assignment of protein clusters, a representative ortholog was randomly selected and analyzed using InterProScan v. 5.23 (68). Orthologous clusters predicted to have either transposase or integrase function were identified. All predicted transposases and integrases were used as queries in BLASTP v. 2.5.0+ searches of translated genome sequences. All hits below a 1e−5 cutoff were identified.

Core and pan-genome estimates were calculated using nonlinear regression (27). A custom script was used to generate 100 permuted replicates of the orthologous cluster matrix from the GET_HOMOLOGUES output (69). The pan-genome rarefaction curve was estimated using the function *n* = *σ*(number of genomes)^γ^ (28). The core genome accumulation curve was estimated using the function *F*(*n*) = κ × exp(−number of genomes/τ) + Ω, where Ω is the estimate of the core genome size (27). The R nls function was used to estimate the free parameters of these equations (70).

The presence/absence matrix generated by GET_HOMOLOGUES was used to determine the core genomes for the *Rathayibacter* genus and each *Rathayibacter* species. A subset of the *Rathayibacter* species, excluding R. festucae and R. caricis, were examined to identify genes absent from the core genome of R. toxicus and present in all other *Rathayibacter* species. Comparisons were executed to identify core genes unique to R. toxicus. Functions of each of the translated sequences were extracted from the InterProScan output and compared to identify functions unique to the core *Rathayibacter* genome (excluding R. toxicus) or the R. toxicus genome.

Significantly enriched clusters in the *Rathayibacter* genus were identified using a one-sided Welch’s *t* test, with a Bonferroni corrected *P* value of 0.01. To account for the difference in the numbers of strains of *Rathayibacter* and other genera (114 and 29, respectively), we randomly subsampled 100 times 29 isolates from *Rathayibacter*. Only clusters that were considered enriched in all 100 subsamples were used in the final analysis. The t.test and p.adjust methods implemented in R were used (70).

The Canberra distance function and Ward’s hierarchical clustering method (ward.D2), implemented in the hclust function of R, was used for hierarchical cluster analysis of the presence/absence matrix (70). Heatmaps were plotted using the gplots package implemented in R (70, 71).

Repeat regions were identified using the RepeatScout software (default settings) (72). Repeats shared between genomes were clustered using CD-HIT-EST v4.6 and on a basis of a threshold of 0.95 sequence identity (73).

GI_SVM was used to identify regions with signatures of horizontal gene transfer (HGT), with settings -N 0.9 -t 1 -k 6 -c 5 (29). The genomic regions with signatures for HGT (mergedRes_auto) were used to query all R. toxicus genome sequences. Those genomic regions with homology (≥75% total coverage and ≥90% identity) to a region not identified as having signatures of HGT and in the genome of another isolate of R. toxicus were filtered out.

### Analyses of gene loci.

The transeq application of EMBOSS was used to translate gene sequences of orthologs present in all sequenced R. toxicus and R. tritici-like isolates (74). MAFFT v 7.305b was used to align the amino acid sequences within each of the orthologous groups (75). The orthologs were further partitioned into sets based on their membership to all isolates, all R. toxicus isolates, only R. toxicus clade A isolates, only R. toxicus clade B isolates, and only R. toxicus clade C isolates. RevTrans was employed to use the amino acid alignments as scaffolds to generate DNA multiple sequence alignments (76). The nuc.div function of the APE R package was used to calculate nucleotide diversity for each orthologous group within each set (77). Using the same process, sets of 100 bootstrap replications of 22, 11, and 7 randomly selected isolates from clade A of R. tritici-like isolates were analyzed for nucleotide diversity. Boxplots were generated, using the ggplot2 R package (78).

Each orthologous group with a nucleotide diversity value of 0 within any clade of R. toxicus and a nucleotide diversity of >0 in the “all isolates” set were identified. Genes from isolates FH138, FH141, and FH100, representatives of clades A, B, and C, respectively, were analyzed using the kaks function of the seqinR R package to measure the ratio of nonsynonymous to synonymous substitutions (*dN*/*dS*) (79)). A chi-squared test was used to test *dN*/*dS* values (80).

CRISPR spacers were identified using the CRISPRFinder web software (81). The CRISPR loci were oriented relative to the Cas-encoding genes. Protospacers were predicted based on BLASTN v 2.5.0+ searches to bacteriophage NCPPB3778 (KX911187.1) (19) with the settings –gapopen 10 –gapextend 2 –reward 1 –penalty -1 –word_size 5, modeled after the CRISPRTarget software (82). BLAST (bit score of >25) was used to group spacers on the basis of similarity.

The CRISPRhit software uses rules previously developed and was written in python and depends on the Biopython package (available at https://www.github.com/osuchanglab/crisprhit) (10, 63).

### Construction of phylogenetic trees and analysis of phyletic patterns.

The species tree was generated on the basis of 353 single-copy core genes that were in all examined genome sequences. Nucleotide sequences were aligned, concatenated, and used as partitioned input. Genome sequences from *Rathayibacter*, *Clavibacter*, and *Leifsonia* were extracted from the NCBI nucleotide databases (on 25 October 2016; see Data Set S1H in the supplemental material). Genomes were required to be of “chromosome” or “scaffold” assembly quality. The phylogenetic tree and the orthologous cluster matrix from the GET_HOMOLOGUES output were used as inputs for the gain loss mapping engine (GLOOME) to determine the predicted gains and losses for each of the phylogenetic clades (83). Four trees were generated, each time substituting for R. toxicus (isolates FH232, FH128, FH137, and FH138). The minimum and maximum values for each branch were determined.

Multiple sequence alignments were performed using MAFFT v. 7.305b (75). Maximum likelihood phylogenetic tree inference was done using RAxML v. 8.2.8 with 100 maximum likelihood tree searches (84). Bootstraps were assessed using the automatic majority rule extended (autoMRE) bootstopping criterion. TreeCollapseCL ver. 4.0 was used to collapse branches with bootstrap support <50% (85). GTRGAMMA and GTRCAT substitution models were used for the species and SNP trees, respectively. Phylogenetic trees were visualized using iTOL v3 (86).
